# Supplementary material for: Nanoscale segregation of channel and barrier claudins enables paracellular ion flux
Source: Nat Commun. 2022 Aug 25;13:4985. doi: 10.1038/s41467-022-32533-4 (PMC9411157; doi:10.1038/s41467-022-32533-4)
Supplement: Supplementary file 1 — Supplementary Information [file 41467_2022_32533_MOESM1_ESM.pdf]

Supplementary Information

Nanoscale segregation of channel and barrier claudins enables paracellular ion flux

Hannes Gonschior[1], Christopher Schmied[1], Rozemarijn Eva Van der Veen[1], Jenny Eichhorst[1], Nina Himmerkus[2], Jörg Piontek[3], Dorothee Günzel[3], Markus Bleich[2], Mikio Furuse[4,5], Volker Haucke[1,6] and Martin Lehmann[1]

[1]Leibniz-Forschungsinstitut für Molekulare Pharmakologie (FMP), 13125 Berlin, Germany.

[2]Institute of Physiology, Christian-Albrechts-University Kiel, 24118 Kiel, Germany.

[3]Clinical Physiology/Nutritional Medicine, Medical Department, Division of Gastroenterology, Infectiology, Rheumatology, Charité – Universitätsmedizin Berlin, 12203 Berlin, Germany.

[4]Division of Cell Structure, National Institute for Physiological Sciences, Okazaki, Aichi 444-8787, Japan.

[5]Department of Physiological Sciences, School of Life Science, SOKENDAI (Graduate University for Advanced Studies), Okazaki, Aichi 444-8585, Japan.

[6]Faculty of Biology, Chemistry and Pharmacy, Freie Universität Berlin, 14195 Berlin, Germany.

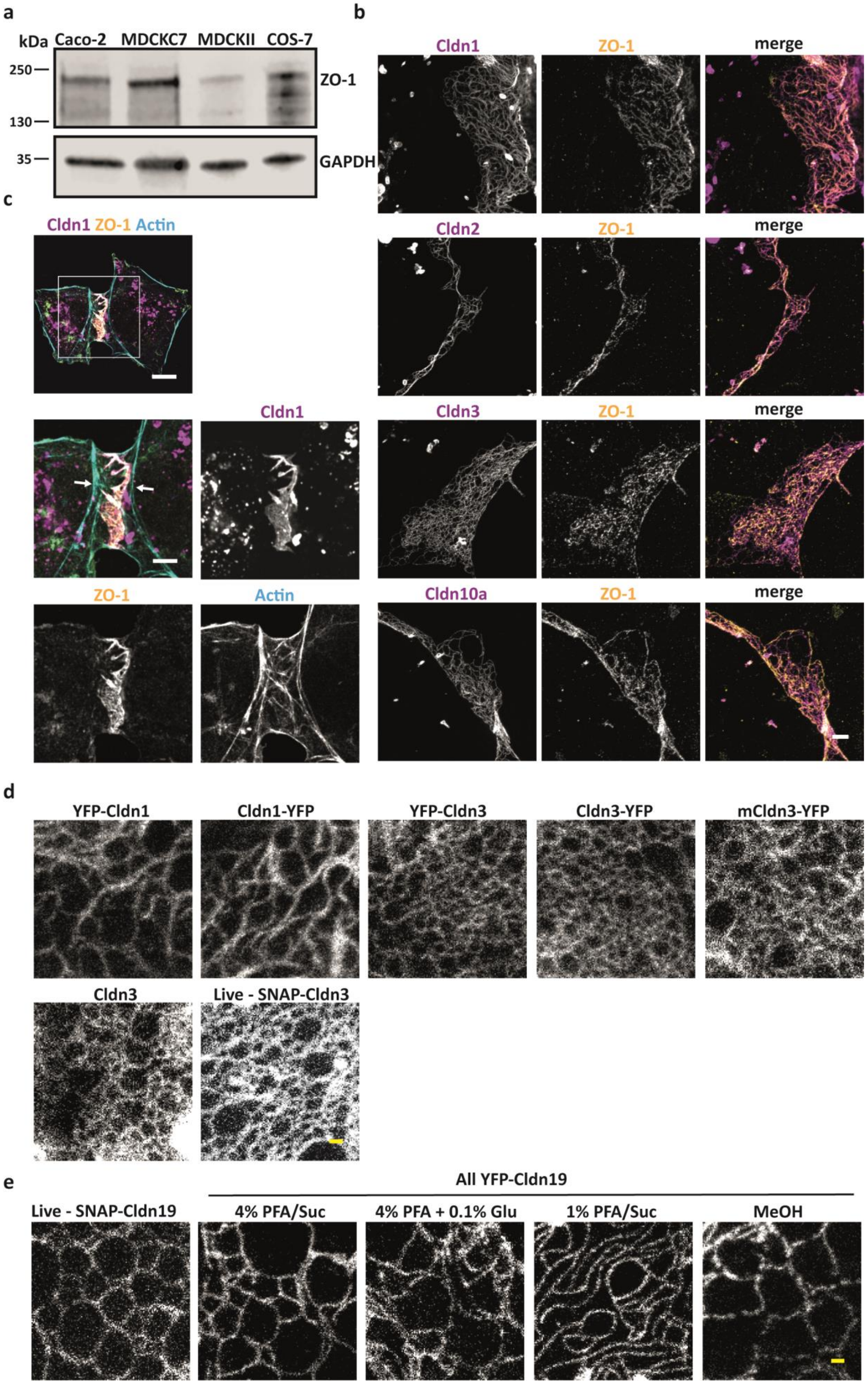
a
kDa
Caco-2
MDCKC7
MDCKII
COS-7
250
130
ZO-1
35
GAPDH
b
Cldn1
ZO-1
merge
Cldn2
ZO-1
merge
Cldn3
ZO-1
merge
Cldn10a
ZO-1
merge
c
Cldn1 ZO-1 Actin
Cldn1
ZO-1
Actin
d
YFP-Cldn1
Cldn1-YFP
YFP-Cldn3
Cldn3-YFP
mCldn3-YFP
Cldn3
Live - SNAP-Cldn3
e
All YFP-Cldn19
Live - SNAP-Cldn19
4% PFA/Suc
4% PFA + 0.1% Glu
1% PFA/Suc
MeOH

Supplementary Fig. 1 | Characterization of COS-7 cells as model cell line for TJ-like meshwork nanoscale imaging. (a) Immunoblotting of whole cell lysates from three epithelial cell lines Caco-2, MDCKC7, MDCKII and from fibroblast-like COS-7 for endogenous ZO-1 (220 kDa) with GAPDH (35 kDa) as loading control. **(b)** Representative STED images of COS-7 cells expressing SNAP-tagged Cldn1, Cldn2, Cldn3 and Cldn10a (magenta; BG-JF646) and immunostained endogenous ZO-1 (yellow; 2nd- AF594). **(c)** Representative confocal images of COS-7 cells overexpressing SNAP-Cldn1 (magenta; BG-JF646), immunostained ZO-1 (yellow; 2nd-AF594) and actin (cyan; Phalloidin AF488). White arrows point at the accumulation of actin in the periphery of the TJ-like meshwork. **(d)** Comparison of TJ-like meshworks in COS-7 formed by overexpressed N- or C-terminally YFP-tagged Cldn1 and Cldn3 as well as C-terminally YFP-tagged murine Cldn3 (mCldn3) and Cldn3 without any tag (2nd-Atto647N) to SNAP-Cldn3 (BG-JF646) imaged in live STED. YFP-tagged claudins were boosted with α-GFP-NB-Atto647N. **(e)** Comparison of differently fixed (4% PFA/Sucrose (Suc), 4% PFA + 0.1% glutaraldehyde (Glu), 1% PFA/Sucrose (Suc), methanol (MeOH)) YFP-tagged Cldn19a (α-GFP-NB-Atto647N) TJ-like meshworks to TJ-like meshwork formed by overexpressed SNAP-tagged Cldn19a (BG-JF646) in COS-7 cells. All representative images derive from 3 independent experiments. Scale bars, 10 µm (overview in c), 5 µm (magnification in c), 1 µm (b), 200 nm (d,e). Source data are provided as a Source Data file.

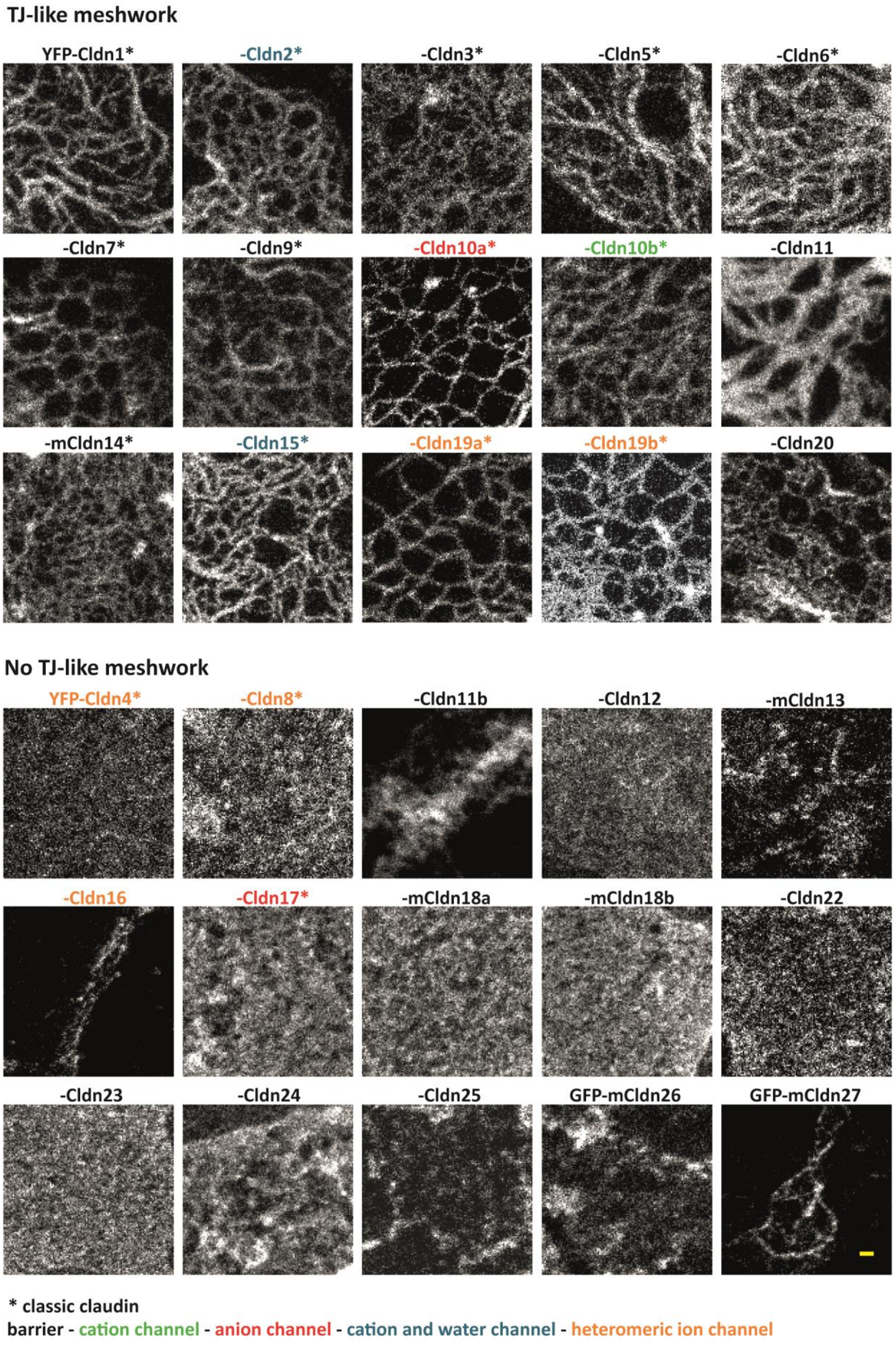
TJ-like meshwork
YFP-Cldn1*
-Cldn2*
-Cldn3*
-Cldn5*
-Cldn6*
-Cldn7*
-Cldn9*
-Cldn10a*
-Cldn10b*
-Cldn11
-mCldn14*
-Cldn15*
-Cldn19a*
-Cldn19b*
-Cldn20
No TJ-like meshwork
YFP-Cldn4*
-Cldn8*
-Cldn11b
-Cldn12
-mCldn13
-Cldn16
-Cldn17*
-mCldn18a
-mCldn18b
-Cldn22
-Cldn23
-Cldn24
-Cldn25
GFP-mCldn26
GFP-mCldn27
* classic claudin
barrier - cation channel - anion channel - cation and water channel - heteromeric ion channel

Supplementary Fig. 2 | TJ-like meshwork screen of all 26 mammalian claudins and their important isoforms expressed in COS-7 cells. Representative STED images of meshwork forming claudins and of non-meshwork forming claudins transfected into COS-7 cells. All analyzed claudins were N-terminally tagged with YFP or GFP and boosted with α-GFP-NB-Atto647N. Shown are representative images from cell-cell overlaps of two transfected cells. Claudins were grouped based on amino acid sequence into classic claudins (labeled with an asterisk *) or function, as barrier (black), cation channel (green), anion channel (red), cation and water channel (blue) and heteromeric ion channel formed by two different claudins (yellow). All representative images derive from 3 independent experiments. Scale bar, 200 nm.

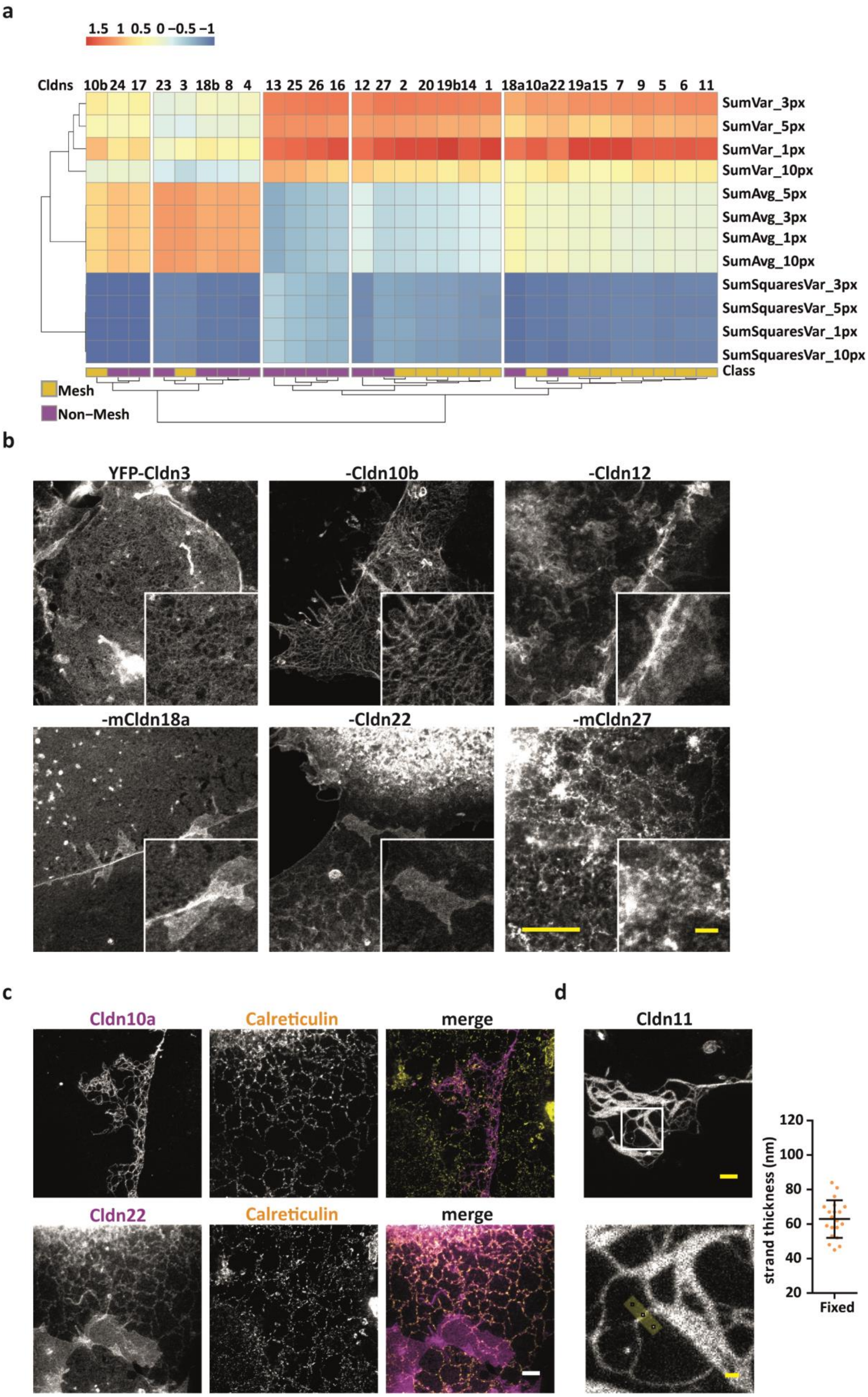

a
1.5 1 0.5 0 −0.5 −1
Cldns 10b 24 17 23 3 18b 8 4 13 25 26 16 12 27 2 20 19b 14 1 18a 10a 22 19a 15 7 9 5 6 11
SumVar_3px
SumVar_5px
SumVar_1px
SumVar_10px
SumAvg_5px
SumAvg_3px
SumAvg_1px
SumAvg_10px
SumSquaresVar_3px
SumSquaresVar_5px
SumSquaresVar_1px
SumSquaresVar_10px
Class
Mesh
Non−Mesh
b
YFP-Cldn3
-Cldn10b
-Cldn12
-mCldn18a
-Cldn22
-mCldn27
c
Cldn10a
Calreticulin
merge
Cldn22
Calreticulin
merge
d
Cldn11
strand thickness (nm)
120
100
80
60
40
20
Fixed

Supplementary Fig. 3 | Haralick texture features analysis of all 26 mammalian claudins and their important isoforms expressed in COS-7 cells. (a) Hierachical clustering of Haralick texture features of all 26 mammalian claudins and isoforms of Cldn10, Cldn18 and Cldn19 expressed in COS-7 cells. All claudins were N-terminally tagged with YFP and boosted with α-GFP-NB-Atto647N. Shown are the SumVariance (SumVar), SumAverage (SumAvg) and the SumSquareVariance (SumSquareVar) over 1, 3, 5, 10 px (20, 60, 100 and 200 nm). Color code represents unit variance scaling as applied to features and euclidean distance with average linkage was used for clustering of columns and rows. The meshwork former class is labeled in yellow and the non-meshwork former class is labeled in magenta. Color code of heatmap represents unit variance scaling and represents number of standard deviations. For more details see Automated TJ-like meshwork analysis in the Methods section. **(b)** Representative overview and magnification images of overlapping regions of misclassified Cldn3 and Cldn10b (dense TJ-like meshworks with a low signal intensity), Cldn12, mCldn18a, Cldn22 and mCldn27 (do not form TJ-like meshwork but show inhomogeneous signal in the overlap and ER localization (Cldn22 with 14/25 cells and Cldn27 with 15/19 cells with ER staining) led to a false interpretation by the algorithm) expressed in COS-7 cells in (a). All claudins were N-terminally tagged with YFP and boosted with α-GFP-NB-Atto647N. **(c)** Representative images of overlapping regions of COS-7 cells transfected with meshwork forming SNAP-Cldn10a or non-meshwork forming SNAP-Cldn22 (both magenta; BG-JF646) and immunostained for the ER marker calreticulin (yellow; 2nd-AF594). **(d)** Representative image of TJ-like meshwork formed by SNAP-Cldn11 (BG-JF646) in COS-7 cells. In the magnification single strands are clearly visible. The yellow rectangle indicates exemplary the area that was taken for FWHM measurements. Full-wide-half-maximum (FWHM) measurement of fixed Cldn11 strands resulted in 63±11 nm. Data represent the mean ± SD. Every data point represents one line profile of total 20 line profiles from one TJ-like meshwork (*n*=20). All representative images derive from 3 independent experiments. Scale bar, 5 µm (overview in b), 2 µm (c), 1 µm (magnification in b, overview in d), 0.2 µm (magnification in d). Source data are provided as a Source Data file.

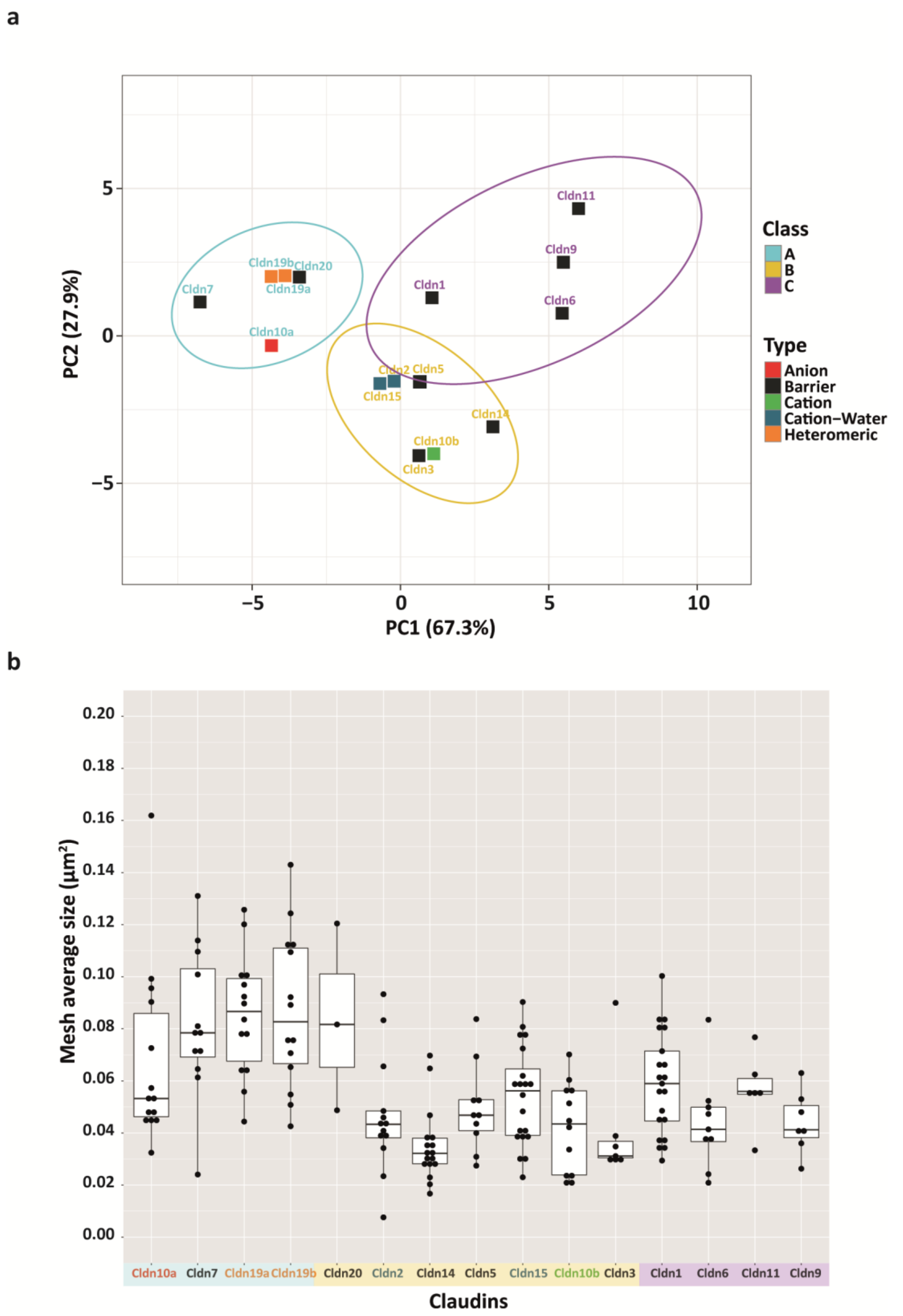

Supplementary Fig. 4 | Principal component analysis of the claudin meshwork formers and their average mesh sizes. (a) Principal component analysis (PCA) data of the analyzed meshwork forming claudins (Class A (cyan), Class B (yellow), Class C (magenta)) (from Fig. 2). Based on a dataset of 15 claudins with 202 total images. Unit variance scaling was applied to features over samples. Single value decomposition with imputation was used for calculating principal components. X and Y axis show principal component 1 and principal component 2 that explain 67.3% and 27.9% of the total

variance, respectively. Prediction ellipses are such that with probability 0.95, a new observation from the same group will fall inside the ellipse. *n*=15 data points. **(b)** Average mesh sizes of all meshwork forming claudins in µm. Claudins are labeled based on their function as barrier (black), cation channel (green), anion channel (red), cation and water channel (blue) and heteromeric ion channel formed by two different claudins (yellow). Data represents median and box of interquartile range and whiskers extend to the largest or smallest value if these values are not further than 1.5 x interquartile range away from the upper or lower quartile values. Every *n* represents one TJ-like meshwork. *n*(Cldn10a)=14; *n*(Cldn7)=12; *n*(Cldn19a)=15; *n*(Cldn19b)=14; *n*(Cldn20)=3; *n*(Cldn2)=13; *n*(Cldn14)=17; *n*(Cldn5)=10; *n*(Cldn15)=20; *n*(Cldn10b)=12; *n*(Cldn3)=7; *n*(Cldn1)=21; *n*(Cldn6)=9; *n*(Cldn11)=6; *n*(Cldn9)=7. From 1-2 independent experiments. Source data are provided as a Source Data file.

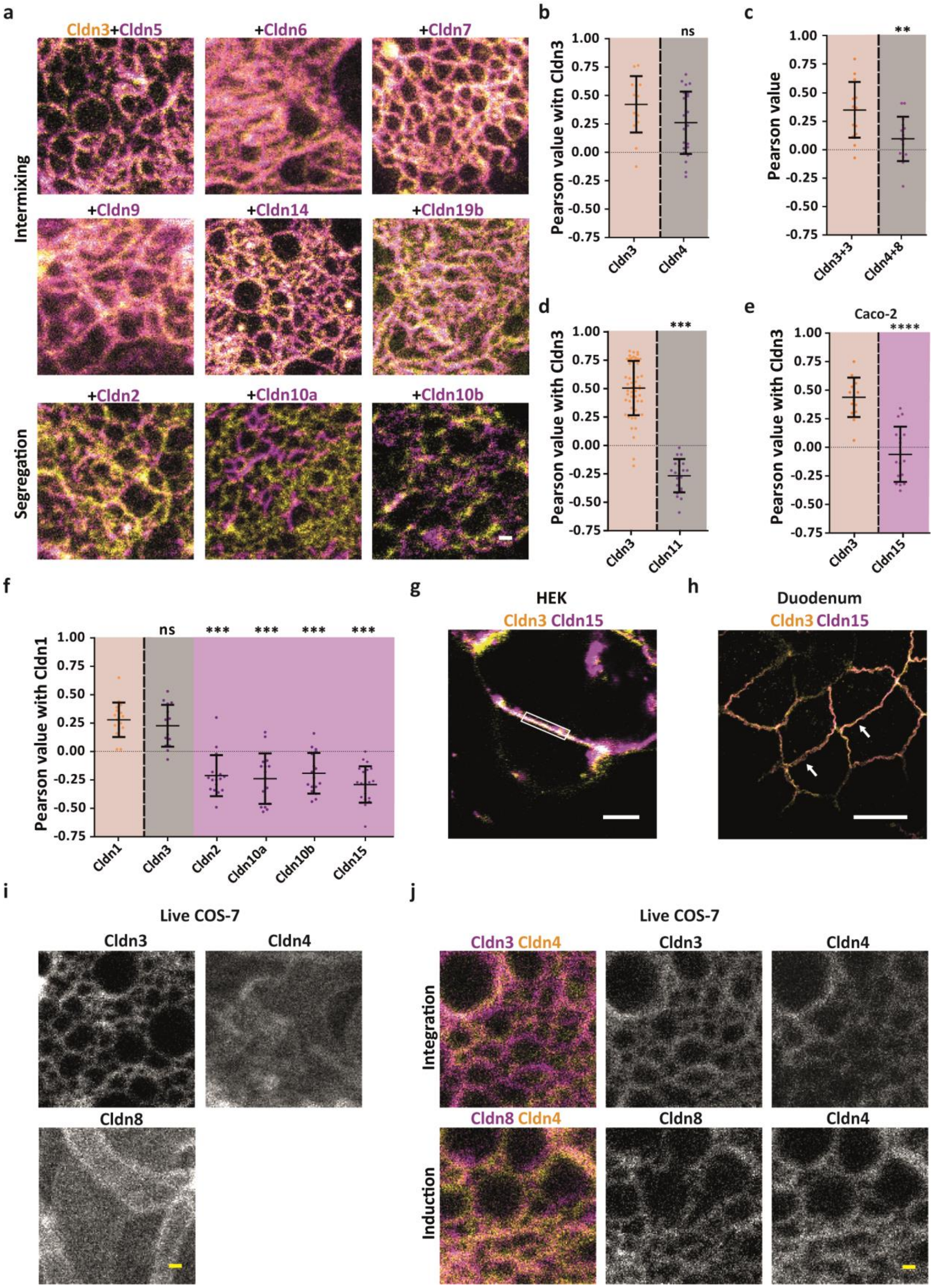

Supplementary Fig. 5 | Meshwork organization of Cldn1, Cldn3 and Cldn4 with other barrier- and channel-forming claudins. (a) Representative STED images of TJ-like meshwork formed by SNAP-Cldn3 (yellow; BG-Atto590) co-expressed with YFP-Cldns (magenta; α-GFP-NB-Atto647N) in COS-7 cells (Pearson correlation analysis is shown in Fig. 3c). **(b)** Pearson correlation analysis of SNAP-Cldn3

co-expressed with YFP-Cldn3 (yellow) and with YFP-Cldn4 (grey). Data represent the mean ± SD. Every *n* represents the Pearson of one TJ-like meshwork. *n*(Cldn3+Cldn3)=15; *n*(Cldn3+Cldn4)=23; from 3 independent experiments; Mann-Whitney test, two-tailed; ns (non-significant) (*P*=0.1099). **(c)** Pearson correlation analysis of SNAP-Cldn3 co-expressed with YFP-Cldn3 a (yellow) and of SNAP-Cldn4 with YFP-Cldn8 (grey). Data represent the mean ± SD. Every *n* represents the Pearson of one TJ-like meshwork. *n*(Cldn3+Cldn3)=15; *n*(Cldn4+Cldn8)=15; from 3 independent experiments; Mann-Whitney test, two-tailed; ** *P*≤0.01 (*P*=0.0095). **(d)** Pearson correlation analysis of SNAP-Cldn3 co-expressed with YFP-Cldn3 (yellow) and of SNAP-Cldn3 (yellow) with YFP-Cldn11 (grey) in COS-7 cells. Data represent the mean ± SD. Every *n* represents the Pearson of one TJ-like meshwork. *n*(Cldn3+Cldn3)= 55; *n*(Cldn3+Cldn11)=21; from 4-5 independent experiments; one-way ANOVA with Dunnett's multiple comparison test; *** *P*≤0.001. **(e)** Pearson correlation analysis of SNAP-Cldn3 co-expressed with YFP-Cldn3 (yellow) and YFP-Cldn15 (magenta) in Caco-2 cells. Data represent the mean ± SD. Every *n* represents the Pearson of one TJ-like meshwork. *n*(Cldn3+Cldn3)=15; *n*(Cldn3+Cldn15)=16; from 3 independent experiments; Mann-Whitney test, two-tailed; **** *P*≤0.0001. **(f)** Pearson correlation analysis of SNAP-Cldn1 co-expressed with YFP-Cldn1 (yellow) and YFP-tagged Cldn3, Cldn2, Cldn10a, Cldn10b and Cldn15 (magenta) in COS-7 cells. Data represent the mean ± SD. Every *n* represents the Pearson of one TJ-like meshwork. *n*(Cldn1+Cldn1)=18; *n*(Cldn1+Cldn)=15; *n*(Cldn1+Cldn2)=17; *n*(Cldn1+Cldn10a)=16; *n*(Cldn1+Cldn10b)=17; *n*(Cldn1+Cldn15)=21; from 3 independent experiments; one-way ANOVA with Dunnett's multiple comparison test, *** *P*≤0.001, ns (non-significant). **(g)** Representative image of HEK cells transfected with Trq2-Cldn3 (yellow) and YFP-Cldn15 (magenta) for spectral FRET analysis. The white box indicates schematically the ROI and the area between claudin expressing HEK cells that was used for the FRET measurements. **(h)** Representative overview image of murine duodenum immunostained for Cldn3 (yellow; 2nd-Atto647N) and Cldn15 (magenta; 2nd-AF594). White arrows point at the formed and with antibodies decorated TJ between neighboring intestinal cells. **(i)** Representative STED images of overlaps formed by SNAP-tagged Cldn3, Cldn4 and Cldn8 (BG-JF646) in living COS-7 cells. **(j)** Representative STED images of overlaps formed by co-overexpressed SNAP-Cldn3 (magenta; BG-JF646) with YFP-Cldn4 (yellow) and SNAP-Cldn8 (magenta; BG-JF646) with YFP-Cldn4 (yellow) in living COS-7 cells. All representative images derive from 3 independent experiments. Scale bars, 5 µm (g, h), 200 nm (a, i, j). Source data are provided as a Source Data file.

a

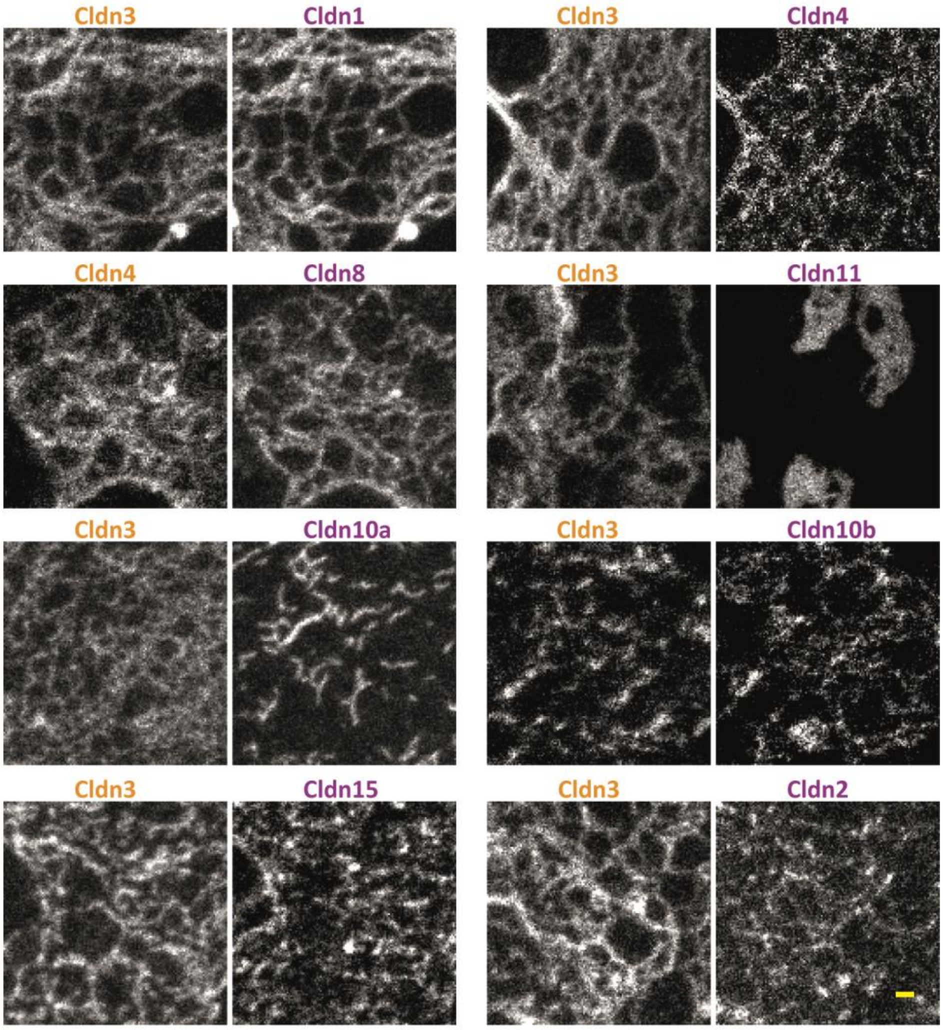

Cldn3
Cldn1
Cldn3
Cldn4
Cldn4
Cldn8
Cldn3
Cldn11
Cldn3
Cldn10a
Cldn3
Cldn10b
Cldn3
Cldn15
Cldn3
Cldn2

b

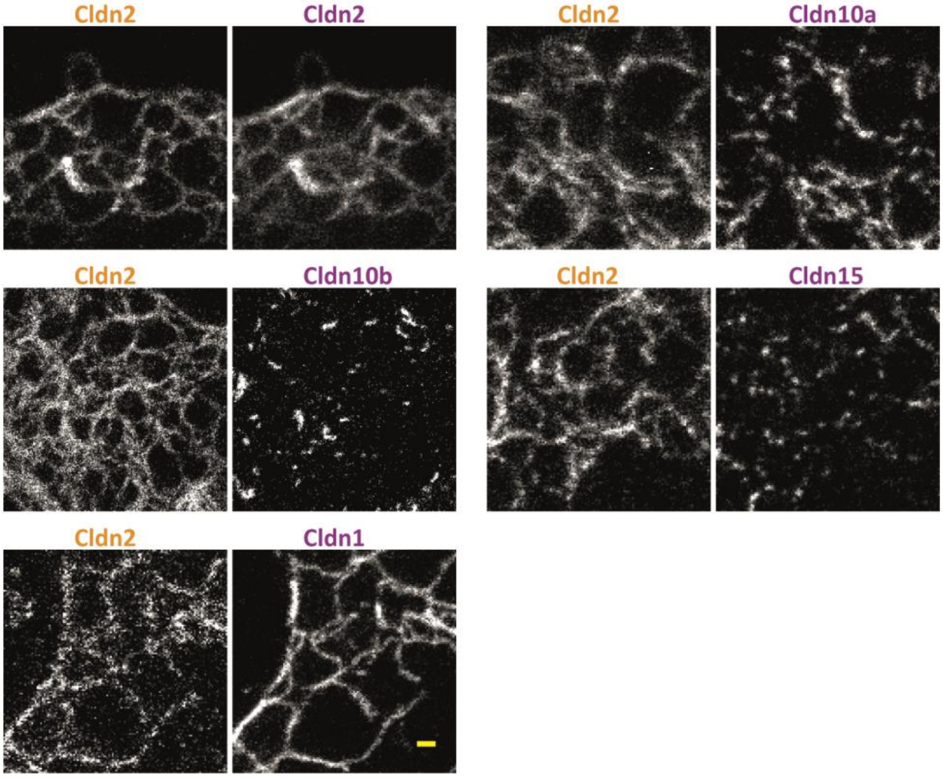

Cldn2
Cldn2
Cldn2
Cldn10a
Cldn2
Cldn10b
Cldn2
Cldn15
Cldn2
Cldn1

Supplementary Fig. 6 | Single channel images from the co-overexpression experiments of Cldn2, Cldn3 and Cldn4 with other channel-forming and barrier-forming claudins. (a) Single channels from the merged STED images shown in Fig. 3b. TJ-like meshworks were formed by SNAP-tagged Cldn3 or Cldn4 (BG-Atto590) in co-overexpression with YFP-tagged Cldn1, Cldn2, Cldn4, Cldn8, Cldn10a, Cldn10b, Cldn11 and Cldn15 (α-GFP-NB-Atto647N). **(b)** Single channels from the merged STED images shown in Fig. 4a. TJ-like meshworks were formed by SNAP-tagged Cldn2 (BG-Atto590) in co-overexpression with YFP-tagged Cldn1, Cldn2, Cldn10a, Cldn10b, and Cldn15 (α-GFP-NB-Atto647N). Scale bars, 200 nm (a, b).

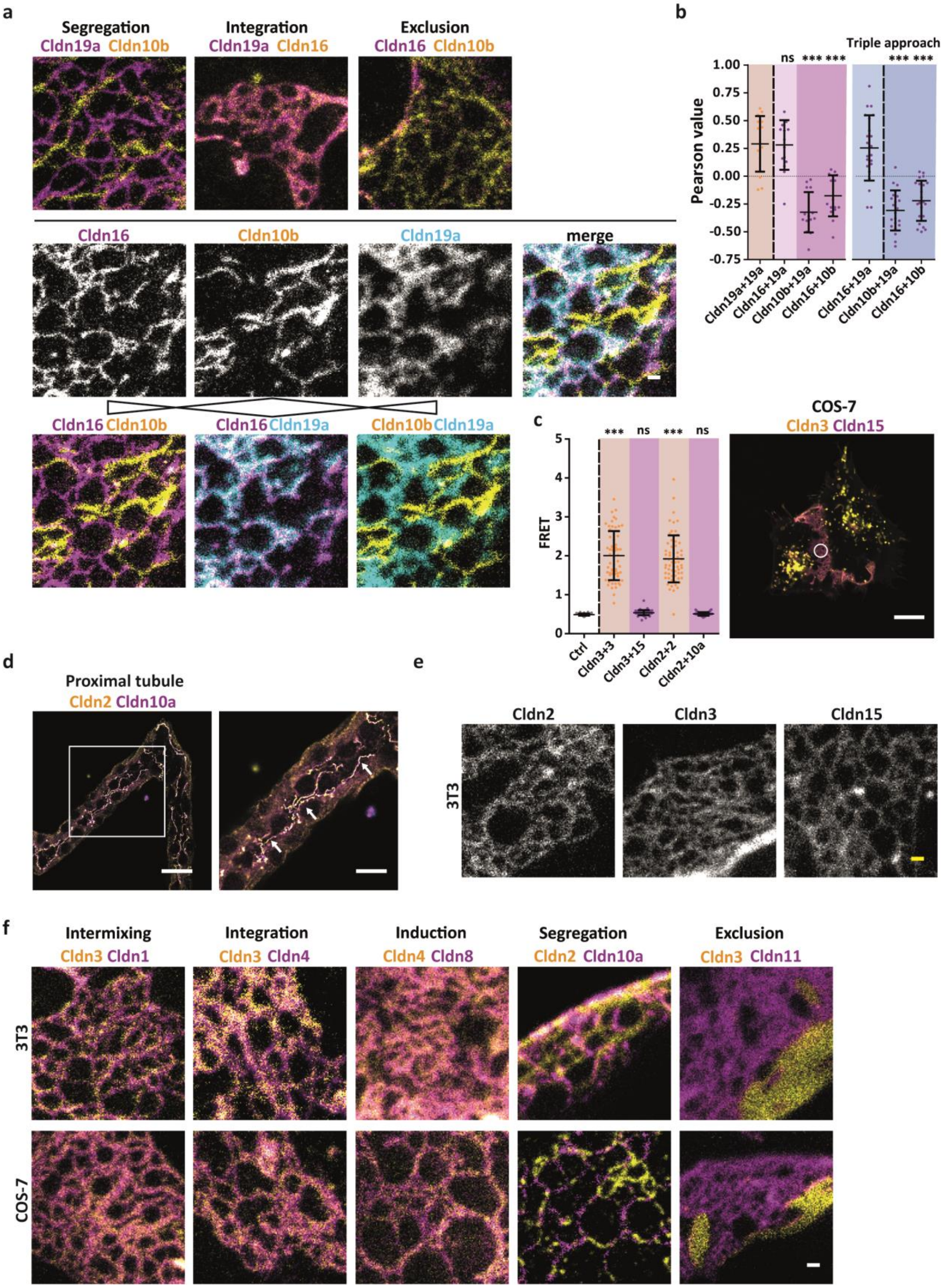

Supplementary Fig. 7 | Meshwork organization of kidney specific expressed claudins in double and triple co-overexpression, FRET in COS-7 cells and TJ-like meshwork in 3T3 cells. (a) Representative STED images of TJ-like meshwork formed by co-overexpression of SNAP-tagged claudins (yellow; BG-Atto590) and YFP-tagged claudins (magenta; α-GFP-NB-Atto647N) and triple overexpression of Halo-

Cldn16 (magenta; CA-JF646), SNAP-Cldn10b (yellow; BG-Atto590) and YFP-Cldn19a (cyan; α-GFP 2nd-Atto542) in fixed COS-7 cells. **(b)** Pearson correlation analysis of co-overexpression (yellow and magenta) and triple overexpression (blue) of Cldn10b, Cldn16 and Cldn19a. Data represent the mean ± SD. Every *n* represents the Pearson of one TJ-like meshwork. *n(*Cldn19a+Cldn19a)=15; *n*(Cldn16+Cldn19a)=15 (double)/22 (triple); *n*(Cldn10b+Cldn19a)=15 (double)/22 (triple); *n*(Cldn16+Cldn10b)=16 (double)/22 (triple); from 3 independent experiments; one-way ANOVA with Dunnett's multiple comparison test; *** $P \leq 0.001$, ns (non-significant). **(c)** Spectral FRET analysis of Trq2-Cldn3 (blank; negative control), Trq2-Cldn3 co-expressed with indicated YFP-tagged Cldn3 (yellow; positive control) or Cldn15 (magenta) and Trq2-Cldn2 co-expressed with YFP-tagged Cldn2 (yellow; positive control) or Cldn10a (magenta) in COS-7 cells. Data represent the mean ± SD. Every *n* represents one cell-cell contact. *n*(negative control)=73; *n*(Cldn3+Cldn3)=61; *n*(Cldn3+Cldn15)=67; *n*(Cldn2+Cldn2)=64; *n*(Cldn2+Cldn10a)=69 from 2 independent experiments; one-way ANOVA with Dunnett's multiple comparison test; *** $P \leq 0.001$, ns (not significant). Representative image of COS-7 cells overexpressing Trq2-Cldn3 (yellow) and YFP-Cldn15 (magenta) for spectral FRET analysis. The white box indicates schematically the area that was used for the FRET measurements. **(d)** Representative overview and magnification image of an isolated mouse proximal tubule immunostained for Cldn2 (yellow; 2nd-Atto647N) and Cldn10a (magenta; 2nd-AF594). White arrows in the magnification point at the formed and labeled TJ between neighboring kidney cells. **(e)** Representative STED images of TJ-like meshworks formed by overexpressed SNAP-tagged Cldn2, Cldn3 and Cldn15 (BG-JF646) in 3T3 fibroblasts. **(f)** Representative STED images of TJ-like meshworks formed by overexpressed SNAP-Cldn3 with YFP-Cldn1 (intermixing), SNAP-Cldn3 with YFP-Cldn4 (integration), SNAP-Cldn8 with YFP-Cldn4 (induction), SNAP-Cldn3 with YFP-Cldn15 (segregation) and SNAP-Cldn3 with YFP-Cldn11 (exclusion) in 3T3 fibroblasts and COS-7 cells (3T3: BG-Atto590 and α-GFP-NB-Atto647N; COS-7: BG-JF646 and α-GFP-NB-Atto594). All representative images derive from 3 independent experiments. Scale bars 20 µm (overview in d), 10 µm (c, magnification in d), 200 nm (a, e, f). Source data are provided as a Source Data file.

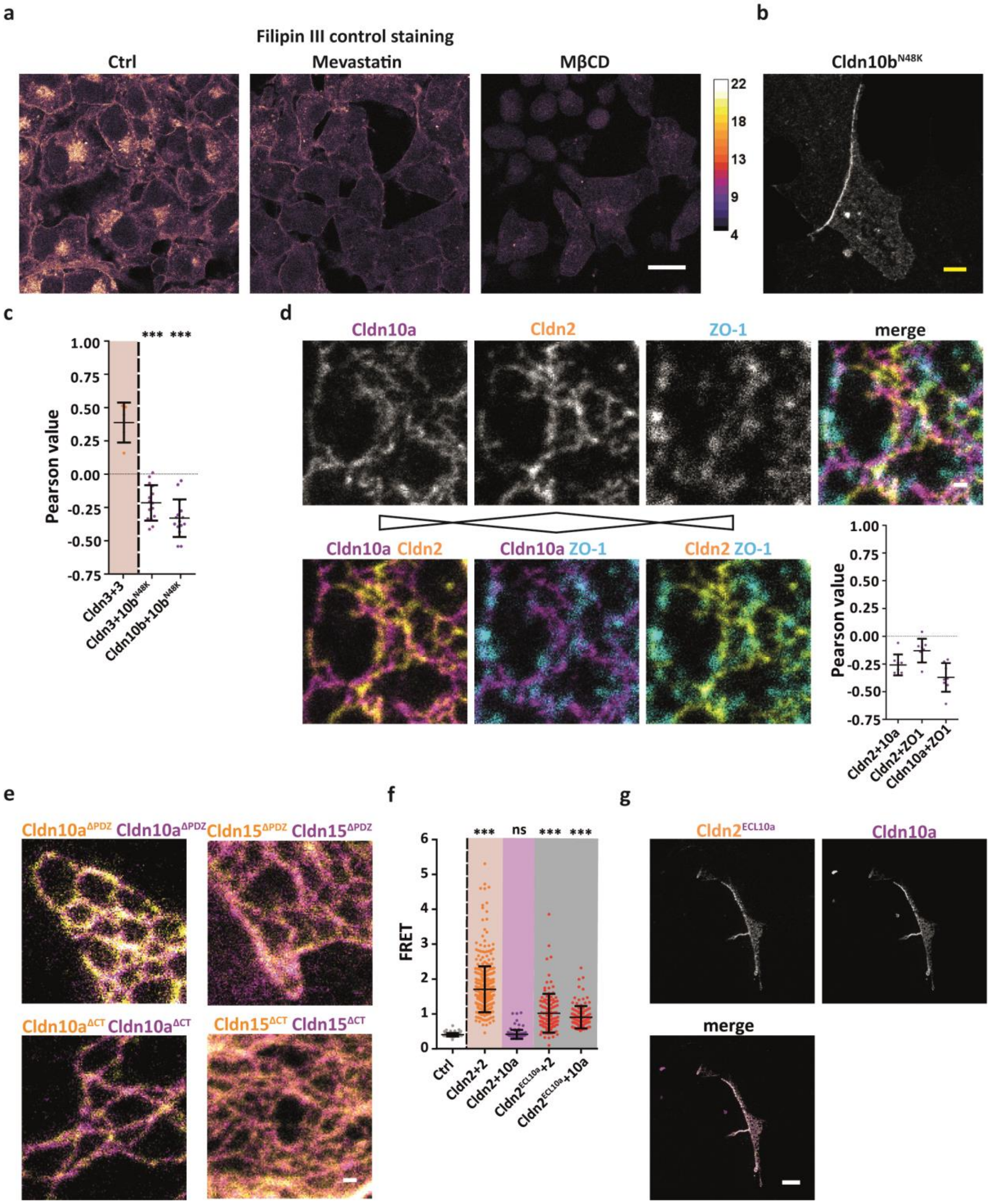

Supplementary Fig. 8 | Claudin segregation is based on the extracellular loops of claudins but not on cholesterol or binding to ZO-1. (a) Representative control staining with Filipin III (specific cholesterol binding, fluorescent) of COS-7 cells treated with either H_2O as ctrl, mevastatin and Methyl-β-cyclodextrin (MβCD) shown in fire color mode (intensity range from 4-22). **(b)** Representative STED image of the overlap of YFP-Cldn10b^{N48K} (α-GFP-NB-Atto647N) expressing COS-7 cells. **(c)** Pearson analysis of SNAP-Cldn3 with YFP-Cldn3 (yellow), SNAP-Cldn3 with YFP-Cldn10b^{N48K} and SNAP-Cldn10b with YFP-Cldn10b^{N48K} co-expressed in COS-7 cells (BG-Atto590; α-GFP-NB-Atto647N). Data represent the mean ± SD. Every *n* represents the Pearson of one TJ-like meshwork.

n(Cldn3+Cldn3)=5; *n*(Cldn3+Cldn10b^{N48K}) =15; *n*(Cldn10b+Cldn10b^{N48K})=15; from 1-3 independent experiments; one-way ANOVA with Dunnett's multiple comparison test; *** $P \leq 0.001$. **(d)** Representative STED image of ZO-1 (cyan; 2nd-Atto542) immunostained in COS-7 cells co-transfected with SNAP-Cldn2 (yellow; BG-Atto590) and YFP-Cldn10a (magenta; α-GFP-NB-647N). Pearson analysis of the combinations Cldn2/ZO-1 (*n*=8), Cldn10a/ZO-1 (*n*=8) and Cldn2/Cldn10a (*n*=8); Data represent the mean ± SD; from one experiment. **(e)** Representative STED images of COS-7 cells co-expressing the PDZ binding motif deletion mutants SNAP-Cldn10a$^{\Delta PDZ}$ (yellow) with YFP-Cldn10a$^{\Delta PDZ}$ (magenta), SNAP-Cldn10a$^{\Delta CT}$ (yellow) with YFP-Cldn10a$^{\Delta CT}$ (magenta), SNAP-Cldn15$^{\Delta PDZ}$ (yellow) with YFP-Cldn15$^{\Delta PDZ}$ (magenta) and SNAP-Cldn15$^{\Delta CT}$ (yellow) with YFP-Cldn15$^{\Delta CT}$ (magenta) (BG-JF646; α-GFP 2nd-AF594). **(f)** Spectral FRET analysis of Trq2-Cldn2 (blank; negative control) or co-expression of Trq2-Cldn2 with YFP-tagged Cldn2 (yellow) and Cldn10 (magenta) and Trq2-Cldn2^{ECL10a} with YFP-tagged Cldn2 and Cldn10a (both red) in HEK cells. Data represent the mean ± SD. Every *n* represents one cell-cell contact. *n*(negative control)=126, one experiment; *n*(Cldn2+Cldn2)=405; *n*(Cldn2+Cldn10a)=115; *n*(Cldn2^{ECL10a}+Cldn2)=114; *n*(Cldn2^{ECL10a}+Cldn10a)=112; all from 4-10 independent experiments; one-way ANOVA with Dunnett's multiple comparison test; *** $P \leq 0.001$, ns (not significant). **(g)** STED image of co-cultured SNAP-Cldn2^{ECL10a} (yellow; BG-Atto590) and YFP-Cldn10a (magenta; α-GFP-NB-647N) expressing COS-7 cells. Shown is an overlap of two COS-7 cells. One cell is expressing SNAP-Cldn2^{ECL10a} and the other cell is expressing YFP-Cldn10a. All representative images derive from 3 independent experiments. Scale bars, 20 µm (a), 2 µm (b, g), 200 nm (d, e). Source data are provided as a Source Data file.

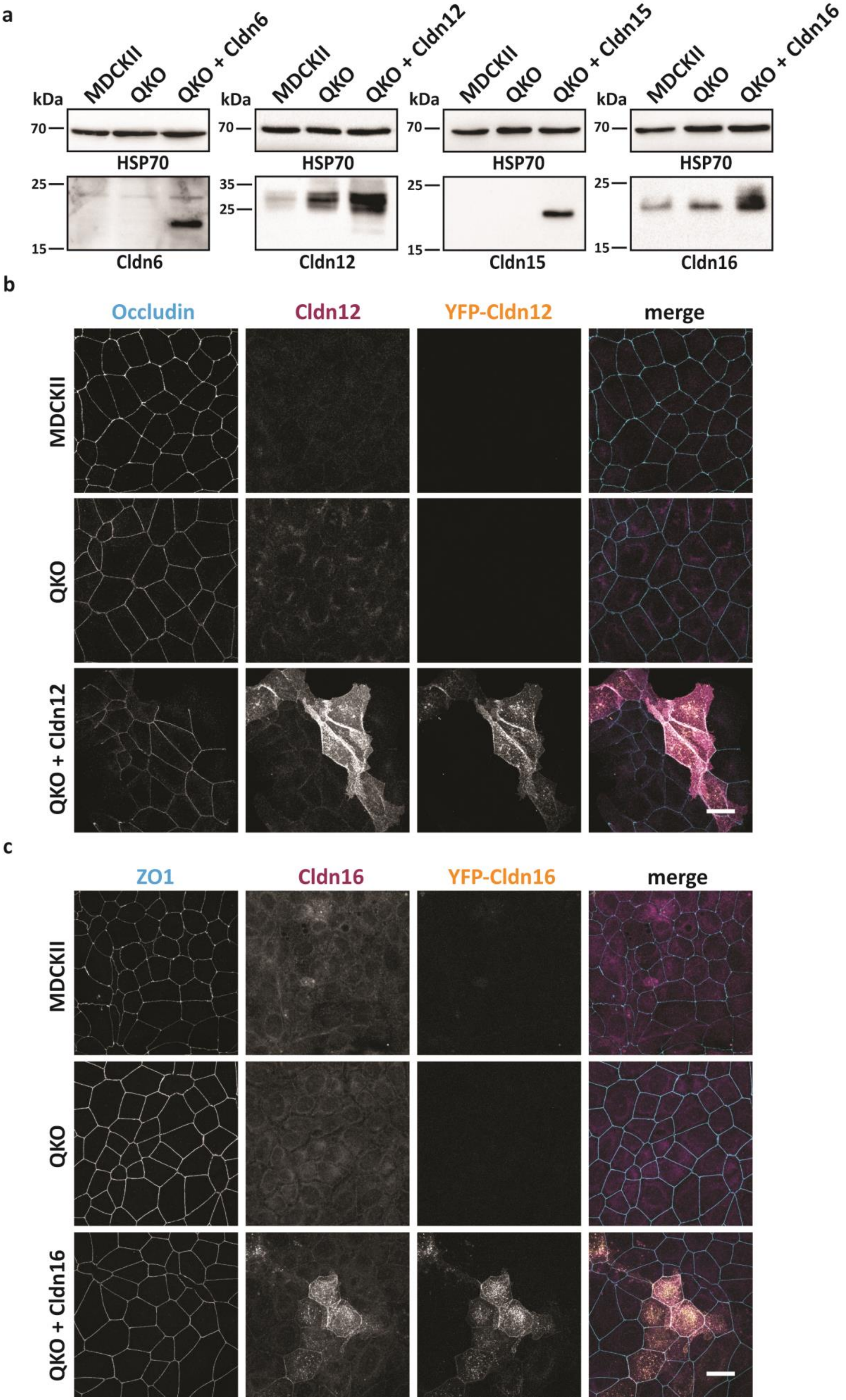
a
MDCKII
QKO
QKO + Cldn6
QKO + Cldn12
QKO + Cldn15
QKO + Cldn16
kDa
70
HSP70
25
15
35
Cldn6
Cldn12
Cldn15
Cldn16
b
Occludin
Cldn12
YFP-Cldn12
merge
MDCKII
QKO
QKO + Cldn12
c
ZO1
Cldn16
YFP-Cldn16
merge
MDCKII
QKO
QKO + Cldn16

Supplementary Fig. 9 | Western Blot and immunofluorescence staining for Cldn6, Cldn12, Cldn15 and Cldn16 in MDCKII and MDCKII QKO cells. (a) Immunoblotting of whole cell lysates from MDCKII, MDCKII QKO and MDCKII QKO transiently expressing untagged Cldn6, Cldn12, Cldn15 or Cldn16 for Cldn6 (23 kDa), Cldn12 (27 kDa), Cldn15 (24 kDa) and Cldn16 (26 kDa). HSP70 (70 kDa) served as loading control. **(b)** Representative confocal images (max. intensity projection) of MDCKII, MDCKII QKO and MDCKII QKO transiently expressing YFP-Cldn12 (yellow; YFP). Cells were immunostained for Occludin (cyan; 2nd-AF594) and Cldn12 (magenta; 2nd-Atto647N). **(c)** Representative confocal images (max. intensity projection) of MDCKII, MDCKII QKO and MDCKII QKO transiently expressing YFP-Cldn16 (yellow; YFP). Cells were immunostained for ZO-1 (cyan; 2nd-AF594) and Cldn16 (magenta; 2nd-Atto647N). All representative images in this figure derive from at least 3 independent experiments. Scale bars, 20 µm (b, c). Source data are provided as a Source Data file.

a

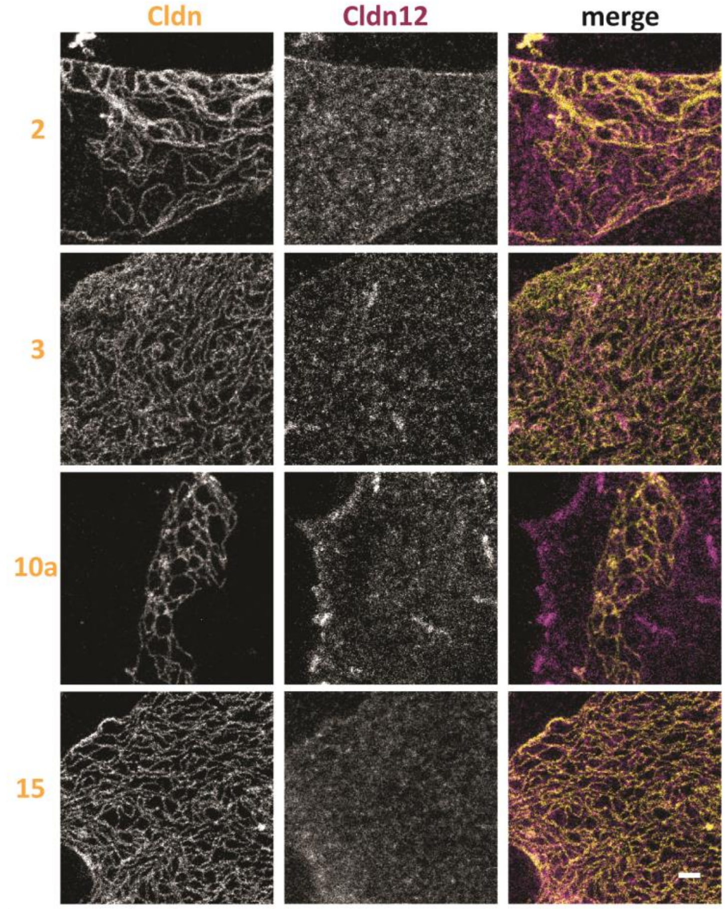

b

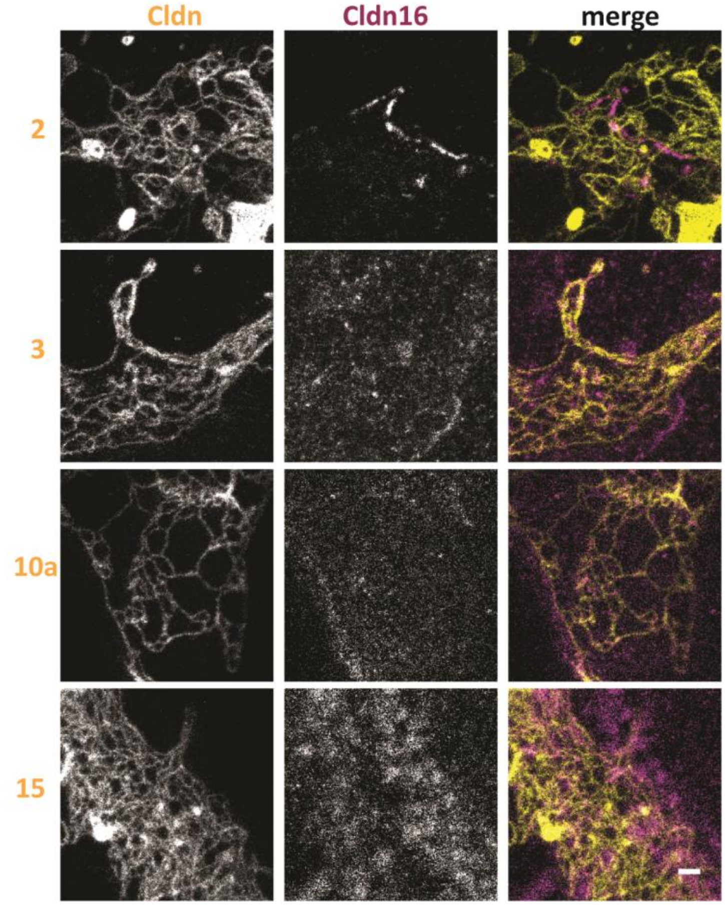

Supplementary Fig. 10 | Co-overexpression experiments of Cldn12 and Cldn16 with Cldn2, Cldn3, Cldn10a and Cldn15 in COS-7 cells. (a) Representative STED images of TJ-like meshworks formed by SNAP-tagged Cldn12 (BG-JF646) in co-overexpression with YFP-tagged Cldn2, Cldn3, Cldn10a and Cldn15 (α-GFP-NB-Atto594) in COS-7 cells. **(b)** Representative STED images of TJ-like meshworks formed by YFP-tagged Cldn16 (α-GFP-NB-Atto594) in co-overexpression with SNAP-tagged Cldn2, Cldn3, Cldn10a and Cldn15 (BG-JF646) in COS-7 cells. All representative images derive from 3 independent experiments. Scale bars, 500 nm (a, b).

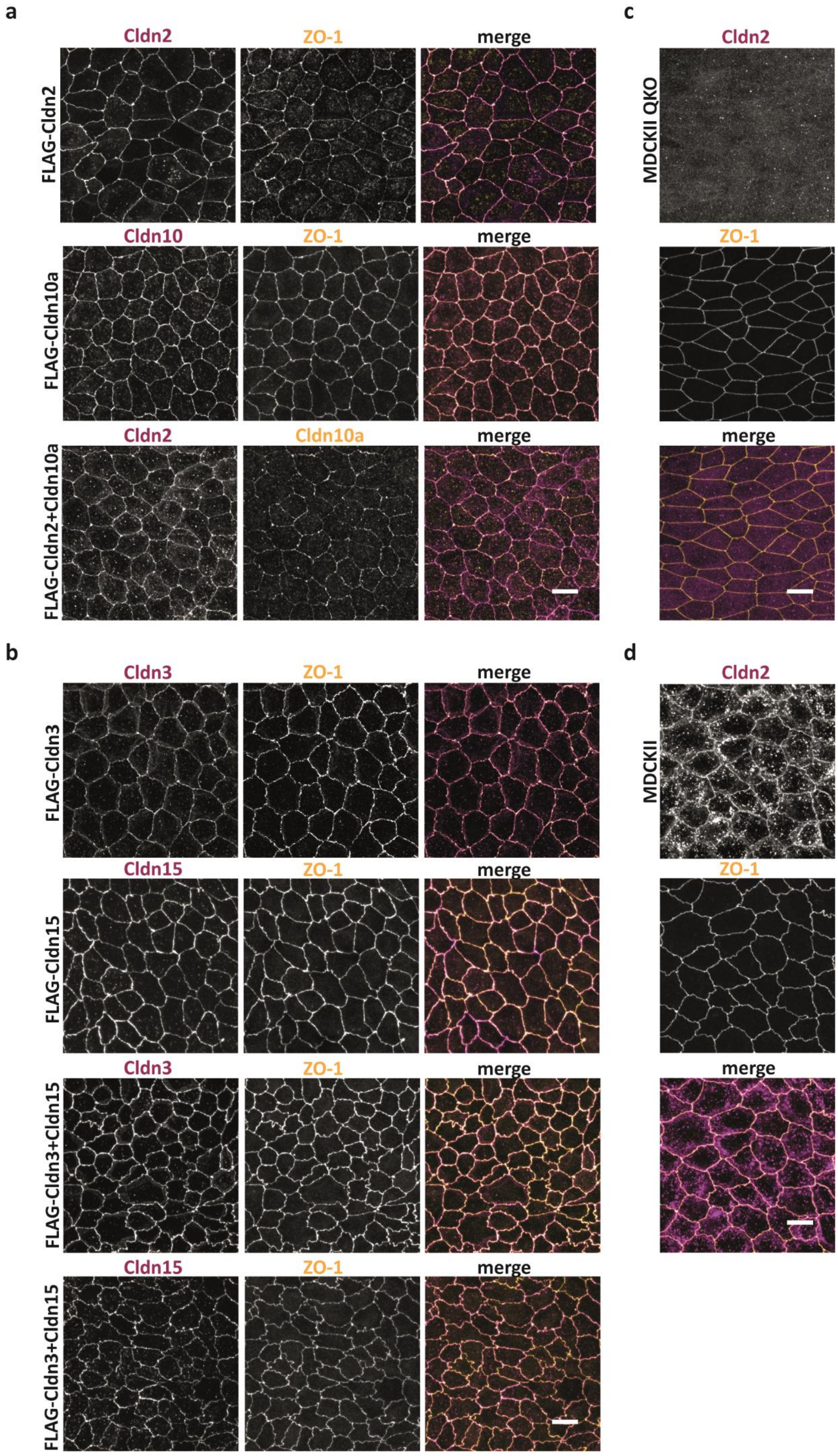
a
Cldn2
ZO-1
merge
FLAG-Cldn2
Cldn10
ZO-1
merge
FLAG-Cldn10a
Cldn2
Cldn10a
merge
FLAG-Cldn2+Cldn10a
c
Cldn2
MDCKII QKO
ZO-1
merge
b
Cldn3
ZO-1
merge
FLAG-Cldn3
Cldn15
ZO-1
merge
FLAG-Cldn15
Cldn3
ZO-1
merge
FLAG-Cldn3+Cldn15
Cldn15
ZO-1
merge
FLAG-Cldn3+Cldn15
d
Cldn2
MDCKII
ZO-1
merge

Supplementary Fig. 11 | Immunofluorescence staining of MDCKII QKO cells stably expressing single claudins and segregating claudin pairs. (a) Representative confocal images (max. intensity projection) of MDCKII QKO stably expressing (sT) FLAG-Cldn2, FLAG-Cldn10a and FLAG-Cldn2+FLAG-Cldn10a. Single claudin expressing cells were stained for Cldn2 (magenta; 2nd-Atto647N) or Cldn10 (magenta; 2nd-Atto647N) and ZO-1 (yellow; 2nd-AF594). Double claudin expressing cells were immunostained for Cldn2 (magenta; 2nd-Atto647N) and Cldn10 (yellow; 2nd-AF594). **(b)** Representative confocal images of MDCKII QKO sT FLAG-Cldn3, FLAG-Cldn15 and FLAG-Cldn3+FLAG-Cldn15. Single and double claudin expressing cells were immunostained for Cldn3 (magenta; 2nd-Atto647N) or Cldn15 (magenta; 2nd-Atto647N) and ZO-1 (yellow; 2nd-AF594). **(c)** Representative confocal images of MDCKII QKO cells immunostained for Cldn2 (magenta; 2nd-Atto647N) and ZO-1 (yellow; 2nd-AF594). **(d)** Representative confocal images of MDCKII cells immunostained for Cldn2 (magenta; 2nd-Atto647N) and ZO-1 (yellow; 2nd-AF594). All representative images derive from 3 independent experiments. Scale bars, 10 µm (a-d).

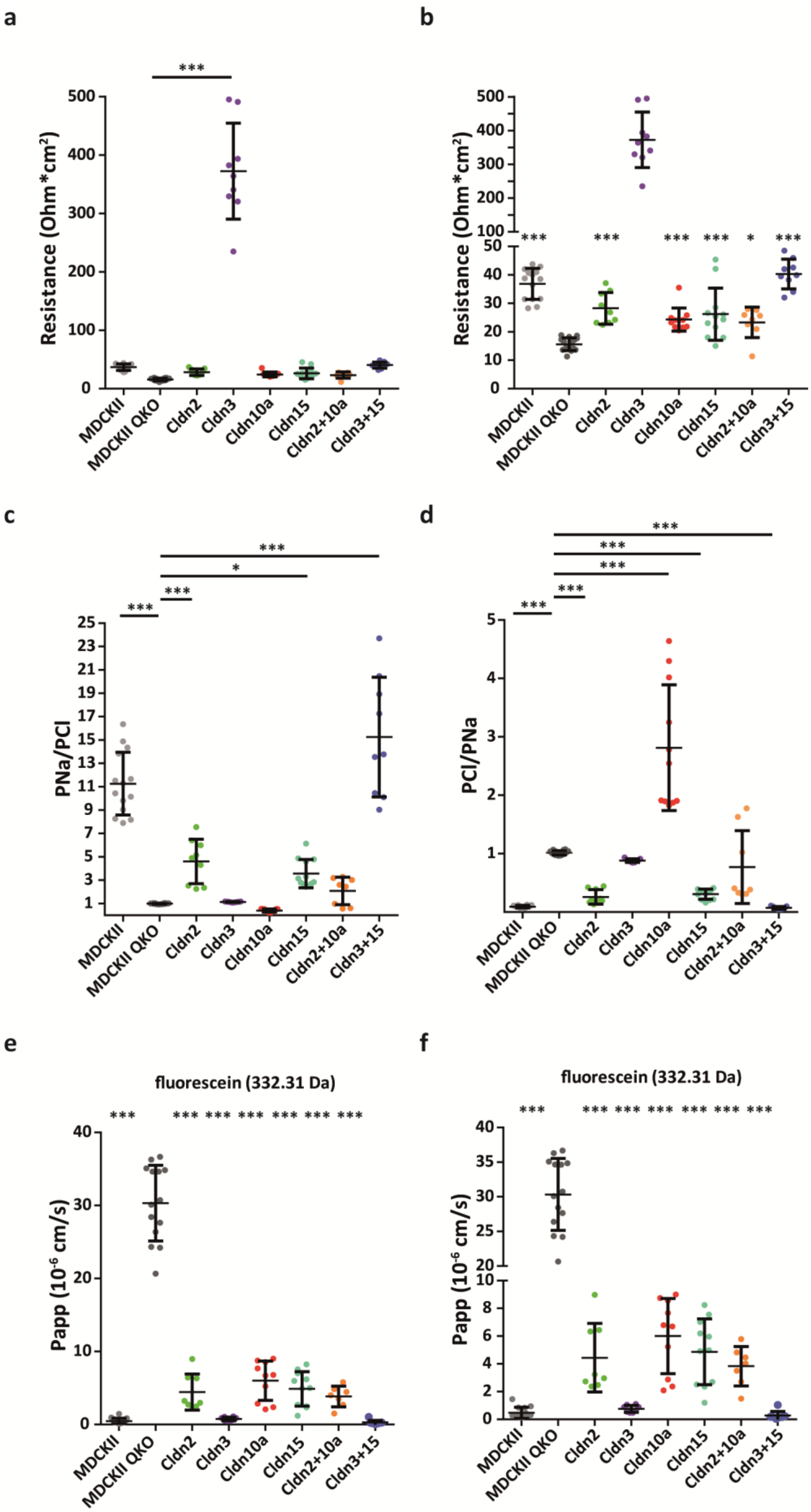
a
b
c
d
e
f
Resistance (Ohm*cm²)
PNa/PCl
PCl/PNa
fluorescein (332.31 Da)
Papp (10⁻⁶ cm/s)
MDCKII
MDCKII QKO
Cldn2
Cldn3
Cldn10a
Cldn15
Cldn2+10a
Cldn3+15
***
*

Supplementary Fig. 12 | Electrophysiological and fluorescein flux measurement of MDCKII, MDCKII QKO and MDCKII QKO cells stably expressing single and multiple claudins. The electrophysiological measurements were performed for MDCKII, MDCKII QKO and MDCKII QKO stably expressing (sT) FLAG-Cldn2, FLAG-Cldn3, FLAG-Cldn10a, FLAG-Cldn15, FLAG-Cldn2+FLAG-Cldn10a and FLAG-Cldn3+FLAG-Cldn15. In every experiment, firstly the trans-epithelial resistance (TER), secondly the dilution potential and lastly the fluorescein flux was measured. Every *n* represents one transwell filter. TER and dilution potential measurements: *n*(WT)=14; *n*(QKO)=16; from 5 independent experiments; *n*(FLAG-Cldn2)=9; *n*(FLAG-Cldn3)=9; *n*(FLAG-Cldn10a)=11; *n*(FLAG-Cldn15)=12; *n*(FLAG-Cldn2+FLAG-Cldn10a)=8; *n*(FLAG-Cldn3+FLAG-Cldn15)=9; from 3 independent experiments. Fluorescein flux measurements: *n*(WT)=14; *n*(QKO)=14; from 5 independent experiments; *n*(FLAG-Cldn2)=8; *n*(FLAG-Cldn3)=8; *n*(FLAG-Cldn10a)=10; *n*(FLAG-Cldn15)=11; *n*(FLAG-Cldn2+FLAG-Cldn10a)=7; *n*(FLAG-Cldn3+FLAG-Cldn15)=9; from 3 independent experiments. One-way ANOVA with Dunnett's multiple comparison test; in (b) FLAG-Cldn3 was not included in statistical comparison; *** $P \leq 0.001$, * $P \leq 0.05$, ns (not significant). **(a)** TER measurements in ohm/cm^2. **(b)** Shown are the results from (a) with a separation in to two segments. **(c)** Dilution potential measurement plotted for the relative permeability as a ratio of Na/Cl. **(d)** Dilution potential measurement plotted for the relative permeability as a ratio of Cl/Na. **(e)** Paracellular permeability (Papp; 10^{-6} cm/s) for fluorescein (332.31 Da). **(f)** Shown are the results from (e) with a separation into two segments. Source data are provided as a Source Data file.

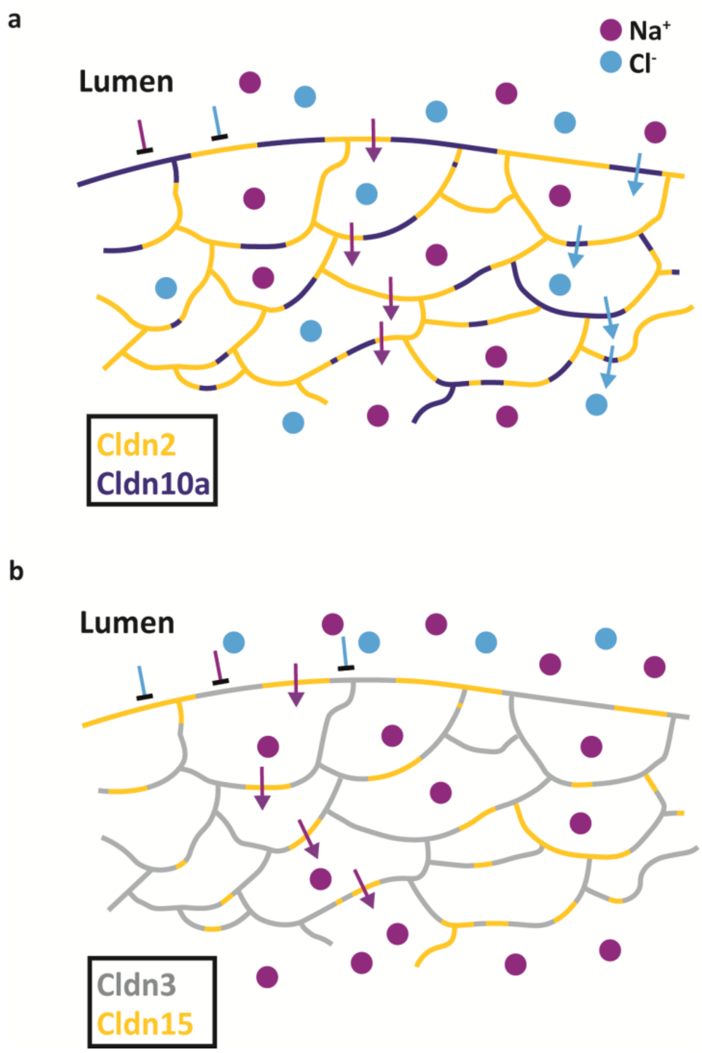

Supplementary Fig. 13 | Refined model of the nano-organization of channel and barrier claudins in the TJ meshwork. (a) Cldn2 (yellow) and Cldn10a (purple) form together a TJ meshwork consisting of several segregating claudin strands enabling the flux of differently charged ions (e.g., Na^+ (magenta) and Cl^- (cyan)) over the TJ meshwork. While the Cldn2 strand patches enable the flux of Na^+ ions, Cldn10a strand patches enable the flux of Cl^- ions. **(b)** Cldn3 (grey) and Cldn15 (yellow) also form a segregating TJ meshwork enabling the flux of only positively charged ions (e.g., Na^+ (magenta)) whereas anions (e.g., Cl^- (cyan)) are not able to cross the formed TJ. While the Cldn15 strand patches enable the flux of Na^+ ions, Cldn3 strand patches act as barrier for anions and cations.
