# Supplementary material for: Nanoscale segregation of channel and barrier claudins enables paracellular ion flux
Source: Nat Commun. 2022 Aug 25;13:4985. doi: 10.1038/s41467-022-32533-4 (PMC9411157; doi:10.1038/s41467-022-32533-4)
Supplement: Supplementary file 3 — Description of additional Supplementary File [file 41467_2022_32533_MOESM3_ESM.pdf]

Descriptions of additional Supplementary Data files

Supplementary Movie 1 | Live-STED movie of a TJ-like meshwork. Single-color STED time series (1 frame/10 s) of a TJ-like meshwork in an overlapping region of living COS 7 cells expressing SNAP-Cldn3 (BG-JF646). White rectangle indicates the area with an initial strand break followed by the fusion of two smaller meshes into a larger mesh. A Gaussian blur with a sigma of 20 nm was applied. Scale bar, 1 µm.

Supplementary Dataset 1 | Table 1: List of all plasmids used in this study; Table 2: List of all primers used in this study; Table 3: List of all restriction enzymes used in this study; Table 4: List of all SNAP and Halo ligands used in this study; Table 5: List of all antibodies used in this study; Table 6: List of all cell dyes used in this study
