# Supplementary material for: Nanoscale segregation of channel and barrier claudins enables paracellular ion flux
Source: Nat Commun. 2022 Aug 25;13:4985. doi: 10.1038/s41467-022-32533-4 (PMC9411157; doi:10.1038/s41467-022-32533-4)
Supplement: Supplementary file 6 — Reporting Summary [file 41467_2022_32533_MOESM6_ESM.pdf]

nature portfolio

Corresponding author(s): Hannes Gonschior, Christopher Schmied, Rozemarijn Eva Van der Veen, Jenny Eichhorst, Nina Himmerkus, Jörg Piontek, Dorothee Günzel, Markus Bleich, Mikio Furuse, Volker Haucke, Martin Lehmann

Last updated by author(s): Aug 19, 2022

Reporting Summary

Nature Portfolio wishes to improve the reproducibility of the work that we publish. This form provides structure for consistency and transparency in reporting. For further information on Nature Portfolio policies, see our Editorial Policies and the Editorial Policy Checklist.

Statistics

For all statistical analyses, confirm that the following items are present in the figure legend, table legend, main text, or Methods section.

n/a	Confirmed	
☐	☒	The exact sample size (n) for each experimental group/condition, given as a discrete number and unit of measurement
☐	☒	A statement on whether measurements were taken from distinct samples or whether the same sample was measured repeatedly
☐	☒	The statistical test(s) used AND whether they are one- or two-sided<br>*Only common tests should be described solely by name; describe more complex techniques in the Methods section.*
☒	☐	A description of all covariates tested
☐	☒	A description of any assumptions or corrections, such as tests of normality and adjustment for multiple comparisons
☐	☒	A full description of the statistical parameters including central tendency (e.g. means) or other basic estimates (e.g. regression coefficient) AND variation (e.g. standard deviation) or associated estimates of uncertainty (e.g. confidence intervals)
☐	☒	For null hypothesis testing, the test statistic (e.g. F, t, r) with confidence intervals, effect sizes, degrees of freedom and P value noted<br>*Give P values as exact values whenever suitable.*
☒	☐	For Bayesian analysis, information on the choice of priors and Markov chain Monte Carlo settings
☒	☐	For hierarchical and complex designs, identification of the appropriate level for tests and full reporting of outcomes
☐	☒	Estimates of effect sizes (e.g. Cohen's d, Pearson's r), indicating how they were calculated

Our web collection on statistics for biologists contains articles on many of the points above.

Software and code

Policy information about availability of computer code

Data collection	The following software were used for data collection:<br>- Confocal and STED images were acquired with a Leica TCS SP8 STED microscope operated by Leica Application Suite X (version 3.5.223225) from Leica Microsystems.<br>- Confocal images were acquired with a Zeiss LSM780 laser scanning confocal microsocope operated by ZEN 2010 B SP1 (version 6.0.0.485) from Carl Zeiss Microscopy.<br>- Western Blots were imaged with ChemiDoc XRS+ (BioRad) controlled by the Image Lab software (version 6.0.1) or with Odyssey Fc imaging system controlled by the Image Studio software (version 5.2).<br>- Dilution potential and flux measurements were performed with a custom-build Ussing-Chamber application at the Institute of Clinical Physiology/Nutritional Medicine, Medical Department, Division of Gastroenterology, Infectiology, Rheumatology, Charité – Universitätsmedizin Berlin.<br>- Fluorescein concentrations were measured with the plate reader Tecan Infinite 200 Pro from Tecan Trading AG.

Data analysis	The following software were used for data analysis:<br>- Microsoft Excel (version 2013) for data storage and processing.<br>- GraphPad Prism (version 5.04) for statistical analysis.<br>- FIJI/ImageJ (version 1.53a/1.53g) for image analysis.<br>- Python (3.7.6) for data analysis.<br>- ClustVis server (biit.cs.ut.ee/clustvis/) for data analysis.<br>- The custom code and example data to reproduce data from Fig.2 is available here: https://doi.org/10.5281/zenodo.7009994

For manuscripts utilizing custom algorithms or software that are central to the research but not yet described in published literature, software must be made available to editors and reviewers. We strongly encourage code deposition in a community repository (e.g. GitHub). See the Nature Portfolio guidelines for submitting code & software for further information.

Data

Policy information about availability of data

All manuscripts must include a data availability statement. This statement should provide the following information, where applicable:

- Accession codes, unique identifiers, or web links for publicly available datasets
- A description of any restrictions on data availability
- For clinical datasets or third party data, please ensure that the statement adheres to our policy

All source data and metadata are reported as excel files for each figure in the supplementary information. All other relevant data and information are available within the paper.

Human research participants

Policy information about studies involving human research participants and Sex and Gender in Research.

Reporting on sex and gender	N/A
Population characteristics	N/A
Recruitment	N/A
Ethics oversight	N/A

Note that full information on the approval of the study protocol must also be provided in the manuscript.

Field-specific reporting

Please select the one below that is the best fit for your research. If you are not sure, read the appropriate sections before making your selection.

[x] Life sciences [ ] Behavioural & social sciences [ ] Ecological, evolutionary & environmental sciences

For a reference copy of the document with all sections, see nature.com/documents/nr-reporting-summary-flat.pdf

Life sciences study design

All studies must disclose on these points even when the disclosure is negative.

Sample size	All sample sizes were chosen based on estimated effect sizes determined from preliminary experiments and published literature. Statistical analyses were used to verify their sufficiency. In general all sample sizes were at least n=3 for each condition, with representative data shown in the manuscript. All experiments in the main manuscript were performed at least three or more times to confirm the results. The exact sample sizes are indicated in the figure legends in the manuscript.
Data exclusions	No samples were excluded from analysis.
Replication	All experiments were repeated independently multiple times to ensure reproducibility of the experimental outcomes as detailed in the legends of the figures. All key experiments were also analyzed for statistical significance.
Randomization	Randomization was not relevant to this study, because all cells and isolated tissue used throughout the study had to be differentially treated and analyzed in parallel to minimize experimental variation. Hence, their identity was known to the investigator.
Blinding	Immunofluorescence images of cells, immunofluorescence images of tissues and electrophysiological mesaurements of cells were captured blindly. Cells for Western Blot were not collected or processed blindly since knowledge of the characteristics of each sample is necessary for data generation.

Reporting for specific materials, systems and methods

We require information from authors about some types of materials, experimental systems and methods used in many studies. Here, indicate whether each material, system or method listed is relevant to your study. If you are not sure if a list item applies to your research, read the appropriate section before selecting a response.

Materials & experimental systems

n/a	Involved in the study	
☐	☒	Antibodies
☐	☒	Eukaryotic cell lines
☒	☐	Palaeontology and archaeology
☐	☒	Animals and other organisms
☒	☐	Clinical data
☒	☐	Dual use research of concern

Methods

n/a	Involved in the study	
☒	☐	ChIP-seq
☒	☐	Flow cytometry
☒	☐	MRI-based neuroimaging

Antibodies

Antibodies used

All antibodies used in this paper are listed in the Supplementary Dataset 1 including antibody dilution, purpose of use and manufacturer details.

The specific details for each primary and secondary antibody including the Research Resource Identifiers (RRID), the link to the manufacturer's website and relevant citations are provided in the following paragraph:

Primary antibodies:
Mouse anti-GFP; Thermo Fisher Scientific; #A-11120; RRID:AB_221568; 1:500 (IF)/1:2000 (WB); verified by relative expression to ensure that the antibody binds to the antigen stated by manufacturer; https://www.thermofisher.com/antibody/product/GFP-Antibody-clone-3E6-Monoclonal/A-11120
Mouse anti-ZO1; Thermo Fisher Scientific; #33-9100; RRID:AB_2533147; 1:100 (IF); knock down validated by manufacturer; https://www.thermofisher.com/antibody/product/ZO-1-Antibody-clone-ZO1-1A12-Monoclonal/33-9100
Rabbit anti-ZO1; Thermo Fisher Scientific; #61-7300; RRID:AB_2533938; 1:100 (IF)/1:1,000 (WB); https://www.thermofisher.com/antibody/product/ZO-1-Antibody-Polyclonal/61-7300
Rabbit anti-Cldn3; Thermo Fisher Scientific; #34-1700; RRID:AB_2533158; 1:100 (IF)/1:500 (WB); verified by relative expression to ensure that the antibody binds to the antigen stated by manufacturer; https://www.thermofisher.com/antibody/product/Claudin-3-Antibody-Polyclonal/34-1700
Rabbit anti-Cldn3-Atto590; this study - details can be found in the methods under "Antibody fluorophore conjugation"; primary antibody is from Thermo Fisher Scientific; #34-1700; RRID:AB_2533158; 1:50 (IF); verified by relative expression to ensure that the antibody binds to the antigen stated by manufacturer; https://www.thermofisher.com/antibody/product/Claudin-3-Antibody-Polyclonal/34-1700
Rabbit anti-Cldn15; Thermo Fisher Scientific; #38-9200; RRID:AB_2533391; 1:100 (IF)/1:500 (WB); we found that the antibody reacts as expected based on morphology and western blots; https://www.thermofisher.com/antibody/product/Claudin-15-Antibody-Polyclonal/38-9200
Rabbit anti-Cldn2; Thermo Fisher Scientific; #51-6100; RRID:AB_2533911; 1:200 (IF)/1:500 (WB); verified by relative expression to ensure that the antibody binds to the antigen stated by manufacturer; https://www.thermofisher.com/antibody/product/Claudin-2-Antibody-clone-MH44-Polyclonal/51-6100
Mouse anti-Cldn2; Thermo Fisher Scientific; #32-5600; RRID:AB_2533085; 1:200 (IF)/1:500 (WB); we found that the antibody reacts as expected based on morphology and western blots; https://www.thermofisher.com/antibody/product/Claudin-2-Antibody-clone-12H12-Monoclonal/32-5600
Rabbit anti-Cldn10; Antibodies-online; #ABIN3183935; 1:200 (IF)/1:500 (WB); we found that the antibody reacts as expected based on morphology and western blots; https://www.antibodies-online.com/antibody/3183935/anti-Claudin+10+CLDN10+C-Term+antibody/
Mouse anti-Cldn10; Thermo Fisher Scientific; #41-5100; RRID:AB_2533510; 1:200 (IF)/1:500 (WB); we found that the antibody reacts as expected based on morphology and western blots; https://www.thermofisher.com/antibody/product/41-5100.html?CID=AFLAP-41-5100
Mouse anti-Occludin; Thermo Fisher Scientific; #33-1500; RRID:AB_2533101; 1:100 (IF)/1:500 (WB); verified by cell treatment by manufacturer; https://www.thermofisher.com/antibody/product/Occludin-Antibody-clone-OC-3F10-Monoclonal/33-1500
Mouse anti-GAPDH; Sigma-Aldrich; #G8795; RRID:AB_1078991; 1:10,000 (WB); we found that the antibody reacts as expected based on western blots; https://www.sigmaaldrich.com/DE/en/product/sigma/g8795
Rabbit anti-Vinculin; Abcam; #ab73412; RRID:AB_1861566; 1:500 (WB); we found that the antibody reacts as expected based on western blots; https://www.abcam.com/vinculin-antibody-epr8185-ab129002.html
Mouse anti-β-actin; Sigma Aldrich; #A5441; RRID:AB_476744; 1:2,000 (WB); we found that the antibody reacts as expected based on western blots; https://www.sigmaaldrich.com/DE/en/product/sigma/a5441
Rabbit anti-SNAP; New England BioLabs Inc.; #P9310S; RRID:AB_10631145; 1:1000 (WB); verified by relative expression to ensure that the antibody binds to the antigen stated by manufacturer; https://international.neb.com/products/p9310-anti-snap-tag-antibody-polyclonal#Product%20Information
Mouse anti-HSP70; Thermo Scientific Fisher; #MA3-006; RRID:AB_325454; 1:5000 (WB); verified by cell treatment by manufacturer; https://www.thermofisher.com/antibody/product/HSP70-Antibody-clone-3A3-Monoclonal/MA3-006

Rabbit anti-Cldn6; Sigma; #191-204;RRID:AB_10611699; 1:1000 (WB); verified by relative expression to ensure that the antibody binds to the antigen stated by manufacturer; https://www.sigmaaldrich.com/DE/en/product/sigma/sab1100902
Rabbit anti-Cldn12; IBL-America; #18801; 1:50 (IF)/1:200 (WB); verified by relative expression to ensure that the antibody binds to the antigen stated by manufacturer; https://www.ibl-america.com/claudin-12-c-anti-mouse-rabbit-igg-affinity-purify/
Mouse anti-Cldn16; was a gift from Prof. Henrik Dimke (University of Southern Denmark, Odense) - Prot-Bertoye et al. 2017 (Am. J. Physiol. Renal. Physiol) tested by immunohistochemistry and immunofluorescence to investigate the distribution of claudin 16 in the human, mouse, and rat kidney.; 1:50 (IF)/1:100 (WB)
Rabbit anti-Calreticulin; Abcam; #ab92516; RRID:AB_10562796; 1:400 (IF); KO verified by manufacturer; https://www.abcam.com/calreticulin-antibody-epr3924-er-marker-ab92516.html
GFP-Booster Atto647N; Chromotek; #gba647-100; RRID:AB_2629215; 1:200 (IF); https://www.ptglab.com/products/GFP-Booster-ATTO647N-gba647n.htm
GFP-Booster Atto594; Chromotek; #gba594-100; RRID:AB_2631387; 1:200 (IF); https://www.ptglab.com/products/GFP-Booster-ATTO594-gba594.htm
GFP-Booster Atto488; Chromotek; #gba488-100; RRID:AB_2631386; 1:200 (IF); https://www.ptglab.com/products/GFP-Booster-ATTO488-gba488.htm

Secondary antibodies:
Donkey anti-mouse AlexaFluor594 Plus; Thermo Fisher Scientific; #A32744; RRID:AB_2762826; 1:200 (IF);
Donkey anti-rabbit AlexaFluor594 Plus; Thermo Fisher Scientific; #A32740; RRID:AB_2762824; 1:200 (IF);
Goat anti-rabbit Atto647N; Active Motif; #15048; 1:200 (IF)
Goat anti-mouse Atto647N; Active Motif; #15058; 1:200 (IF)
Donkey anti-mouse Atto542; this study - details can be found in the methods under "Antibody fluorophore conjugation"; 1:200 (IF)
Donkey anti-rabbit Atto542; this study - details can be found in the methods under "Antibody fluorophore conjugation"; 1:200 (IF)
Goat anti-rabbit Alexa Fluor 488; Thermo Fisher Scientific; #A-11008; RRID:AB_143165; 1:200 (IF)
Donkey anti-rabbit; Jackson Immuno Reasearch Ltd.; #711-005-152; RRID:AB_2340585
Donkey anti-mouse; Jackson Immuno Reasearch Ltd.; #715-005-151; RRID:AB_2340759
Goat anti-mouse HRP conjugated; Jackson Immuno Reasearch Ltd.; #115-035-003; RRID:AB_10015289; 1:2000 (WB)
Goat anti-rabbit HRP conjugated; Jackson Immuno Reasearch Ltd.; #111-035-003; RRID:AB_2313567; 1:2000 (WB)
Donkey anti-mouse IRDye 800CW conjugated; LI-COR; #926-32212; RRID:AB_621847; 1:10,000 (WB)
Donkey anti-rabbit IRDye 800CW conjugated; LI-COR; #926-32213; RRID:AB_621848; 1:10,000 (WB)

Validation

All antibodies used for immunoblotting were validated by including appropriate weight markers and determining if the protein band had the expected molecular weight. For antibodies used for immunofluorescence, antibody specificity was tested by adding positive and negative controls and checking their staining patterns according to what has been published. Details are listed in the section "Antibodies used" above.

Eukaryotic cell lines

Policy information about cell lines and Sex and Gender in Research

Cell line source(s)

HEK293, HEK293T, COS-7, MDCKII and Caco-2 cells were obtained from ATCC. MDCKC7 cells were kindly provided by Lorena Suarez Artiles from the Mertins Lab (MDC Max-Delbrück-Center for Molecular Medicine, Berlin). MDCKII quintuple claudin knock-out (QKO) cells were a kind gift from the lab of Mikio Furuse (National Institute for Physiological Sciences, Okazaki). 3T3 cells were obtained from DSMZ.

Authentication

Cell lines from ATCC and DSMZ are regularly authenticated by STR profiling and were used by us without further authentication. Cell lines obtained from other sources were not authenticated by us.

Mycoplasma contamination

Cell lines were not contaminated and were regularly tested for mycoplasma contamination.

Commonly misidentified lines
(See ICLAC register)

No commonly misidentified cell lines were used.

Animals and other research organisms

Policy information about studies involving animals; ARRIVE guidelines recommended for reporting animal research, and Sex and Gender in Research

Laboratory animals

Mus musculus; C57BL/6J wild type control. Adult animals were used (12 weeks of age). Mice were housed under standardized conditions (12 h light/dark cycle; 22–24 °C temperature; 55% ± 15% humidity ad libitum access to standard diet and water)

Wild animals

No wild animals were used in this study.

Reporting on sex

The sex of the the animals was not considered in study design and a sex- and gender-based analysis was not performed in this study.

Field-collected samples

No field collected samples were used in the study.

Ethics oversight

Mouse experiments were performed in accordance with the German law on animal protection and approved by the local authorities: the Landesamt für Gesundheit und Soziales (LAGeSo) Berlin with their permission under the license T-025/16 and the "Ministerium für Landwirtschaft, Umwelt und ländliche Räume" in Schleswig-Holstein (MELUND SH) with their permission under the animal ethics

protocol number V312-72241.121-2.

Note that full information on the approval of the study protocol must also be provided in the manuscript.
